# MUC15 inhibits cancer metastasis via PI3K/AKT signaling in renal cell carcinoma

**DOI:** 10.1038/s41419-020-2518-9

**Published:** 2020-05-07

**Authors:** Yangyang Yue, Ke Hui, Shiqi Wu, Mengzhao Zhang, Taotao Que, Yanan Gu, Xinyang Wang, Kaijie Wu, Jinhai Fan

**Affiliations:** 1 Department of Urology, First Affiliated Hospital of Xi’an Jiaotong University, Xi’an, 710061 China; 2 Department of Hepatobiliary Surgery, First Affiliated Hospital of Xi’an Jiaotong University, Xi’an, 710061 China; 3 Department of Neurosurgery, Cancer Hospital of the Chinese Academy of Medical Sciences, Beijing, 100021 China

**Keywords:** Tumour biomarkers, Cell invasion

## Abstract

Patients with renal cell carcinoma (RCC) often develop distant metastasis and the specific molecular mechanism remains poorly understood. In our study, we demonstrated that MUC15, a subtype of mucins family, could suppress the progression of RCC by inhibiting PI3K/AKT signaling. Firstly, we observed that MUC15 was notably decreased in RCC compared to normal tissue. Furthermore, we showed that MUC15 could negatively modulate the migration and invasion of RCC in vitro and in vivo. Mechanistically, we found that knocking-down of MUC15 could active the PI3K/AKT signaling by increasing the AKT phosphorylation and subsequently increase the mRNA and protein expression of MMP2 and MMP9. Interruption of the AKT pathway with the specific inhibitor LY294002 could reverse the expression of MMPs. Therefore, our study clarify the novel function of MUC15 in RCC, which may provide a new sight to diagnose and prevent RCC metastasis.

## Introduction

Renal cell carcinoma (RCC) is one of the common malignancies in urological system. There are an estimated 73,820 new cases and 14770 deaths from kidney and renal pelvic cancer in United States in 2019^1^. Major subtypes of RCC are clear cell RCC (ccRCC), papillary RCC (pRCC) and chromophobe RCC (chRCC)^2^, and ccRCC is approximately accounted of 75%^3^. Most of the familial^4^ and about 60% sporadic ccRCC have mutations or deletions in the von Hippel-Lindau (VHL) gene located at 3p(p3.25)^5^. Up to 20% of newly diagnosed patients have developed distant metastasis^6^, and a significant percent about 30% of patients with located cancer have occult metastasis^7–9^. Increased understanding of molecular of metastatic RCC is contributed to the diagnosis and treatment.

MUC15, an important subtype of mucins (MUC) family which are high molecular weight glycoproteins and produced by epithelial cells^10^, has been reported to inhibit tumor proliferation and metastasis in hepatocellular carcinoma^11^ and suppresses invasion of trophoblast-like cells in vitro^12^. In contrast, MUC15 plays an oncogenic role in colon cancer^13,14^, papillary thyroid carcinoma^15,16^. Obviously, the role of MUC15 in cancer development is still confusing, and the expression and function of MUC15 in RCC are yet completely unknown.

In this study, we were the first to confirm that MUC15 was notably decreased in RCC compared to normal tissue. In addition, we demonstrated that MUC15 could suppress the migration and invasion of RCC through PI3K/AKT signaling in vitro and in vivo. Therefore, our study offers more understanding about the mechanism of RCC metastasis, in which MUC15 might be a potential target for RCC diagnosis and treatment.

## Results

### MUC15 is downregulated in human RCC tissues

**Fig. 1:**
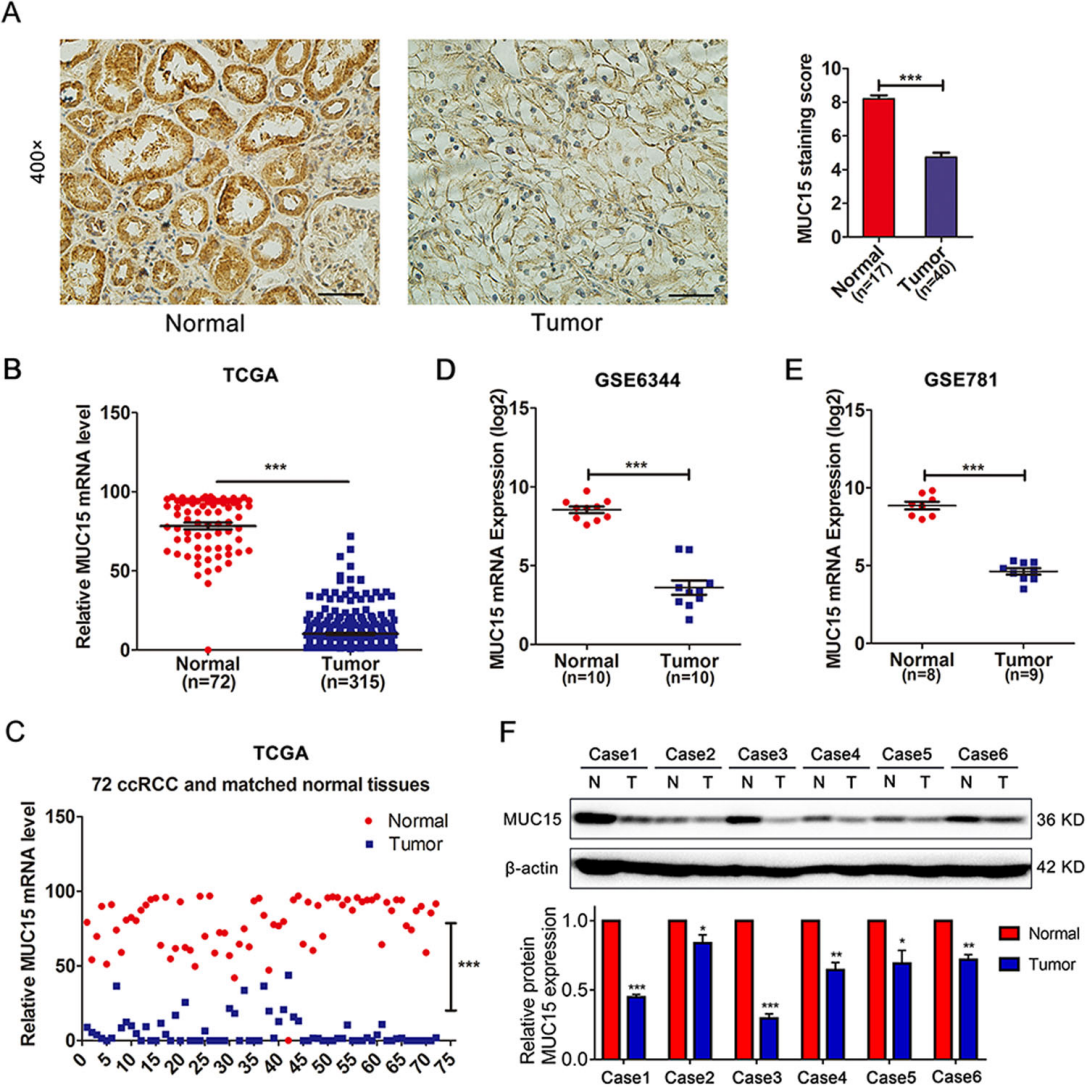
Expression of MUC15 in RCC and normal kidney tissues. **a** Immunohistochemistry staining of MUC15 in normal kidney tissues (*n* = 17) and RCC tissues (*n* = 40). The scale bar is 50 μm. **b** MUC15 mRNA expression in normal kidney tissues (*n* = 72) and RCC tissues (*n* = 315) from TCGA database. **c** MUC15 mRNA expression in 72 paired normal kidney and RCC tissues. **d** MUC15 mRNA expression in matched normal kidney and RCC tissues (*n* = 10) from GEO database (GSE6344). **e** MUC15 mRNA expression in not matched normal kidney (*n* = 8) and RCC tissues (*n* = 9) from GEO database (GSE781). **f** Western blot analysis of MUC15 protein expression level in human normal and renal cancer tissues, the quantification analysis was shown below. β-actin was used as a loading control (*N* = 3).

To explore the expression level of MUC15 in RCC tissues, we performed immunohistochemical staining in the clinical samples including 40 cases of RCC and 17 cases of normal renal tissues. As shown in Fig. 1a, we found that the protein expression of MUC15 was significantly downregulated in RCC tissues compared to normal tubular epithelium (***P < 0.001). Moreover, based on analyzing RNA-sequence in cohort of no-paired samples (315 cases RCC tissues and 72 cases normal kidney tissues) and 72 paired samples from the The Cancer Genome Atlas (TCGA), we could concluded that MUC15 mRNA expression level is obviously lower in RCC tissues (Fig. 1b, c, ***P < 0.001). In addition, from the analysis of public microarray datasets GSE6344 and GSE781, we could also get the same conclusion (Fig. 1d, e, ***P < 0.001). Similar result was observed in human normal and renal cancer tissues by Western blotting assay (Fig. 1f). However, there was no significant correlation between MUC15 and tumor grades or stages (Supplementary Fig. 1A–D), and we also failed to observe a significant correlation between MUC15 expression and overall survival (OS) or disease free survival (DFS) of RCC patients (Supplementary Fig. 1E, F).

### MUC15 suppressed RCC cell migration and invasion in vitro

**Fig. 2:**
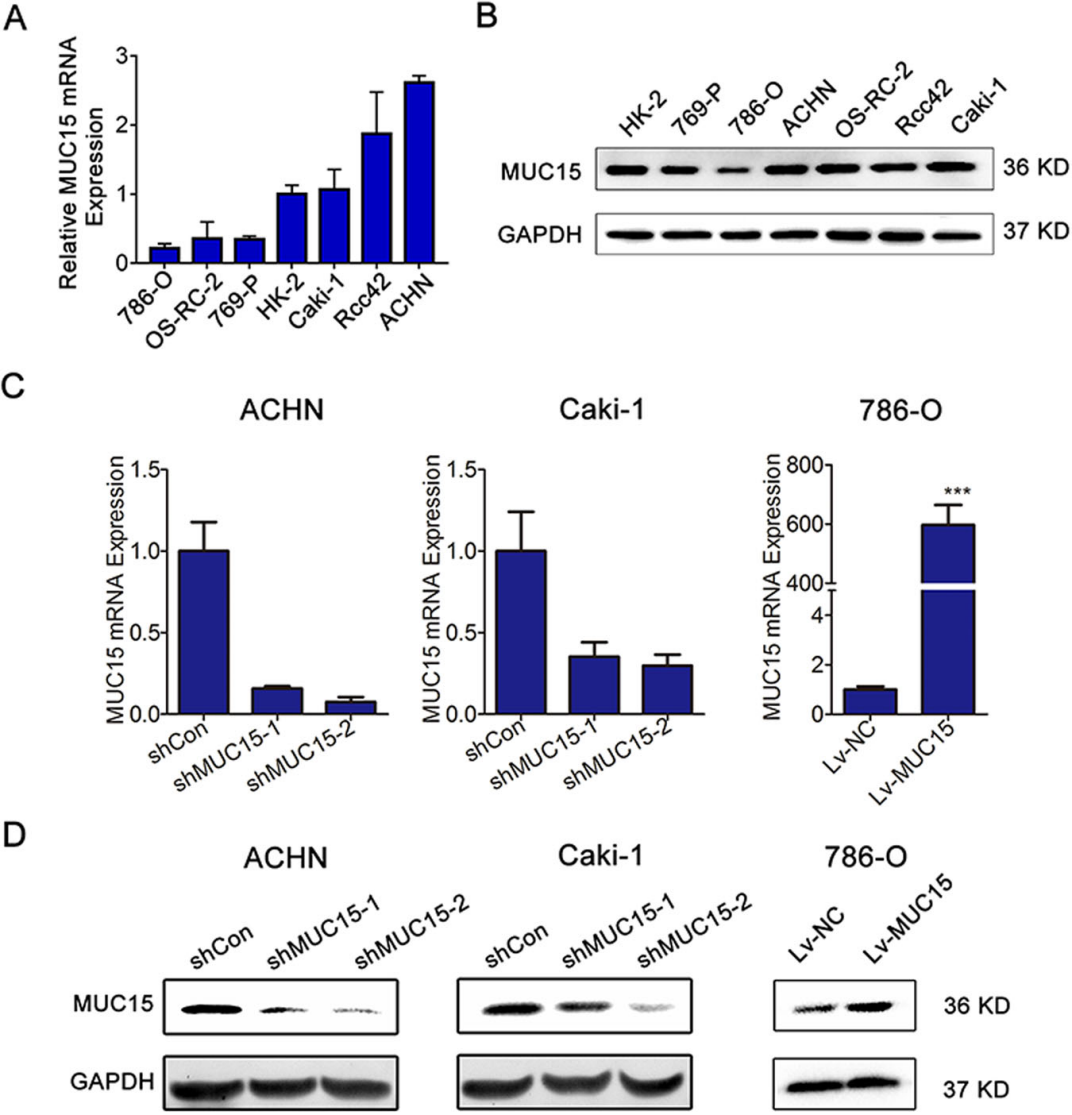
Expression of MUC15 in RCC cell lines and establishment of sublines. **a**, **b** Quantitative real-time RT-PCR and western blot analysis of MUC15 expression level in human normal and renal cancer cell lines (*N* = 3). **c**, **d** Quantitative real-time RT-PCR and western blotting analysis of MUC15 mRNA expression in ACHN or Caki-1 cell lines transfected with MUC15 shRNAs and shControl, and 786-O cell line infected with MUC15 lentivirus and negative control. 18S was applied as the endogenous control for quantitative real-time RT-PCR, and GAPDH was used as a loading control for western blotting assay (*N* = 3).

We also examined the expression level of MUC15 in different RCC cell lines, as shown in Fig. 2a, b, compared to HK-2 cell line, Caki-1, ACHN and Rcc42 cells had relatively higher MUC15 mRNA expression, while ACHN, Caki-1 and OS-RC-2 cells had relatively higher MUC15 protein expression among all tested RCC cell lines. In contrast, MUC15 was lower in 786-O cell lines both in mRNA and protein expression level. Therefore, we successfully generated the stable ACHN and Caki-1 sublines with endogenous MUC15 knockdown and 786-O subline with ectopic MUC15 overexpression (Fig. 2c, d).

**Fig. 3:**
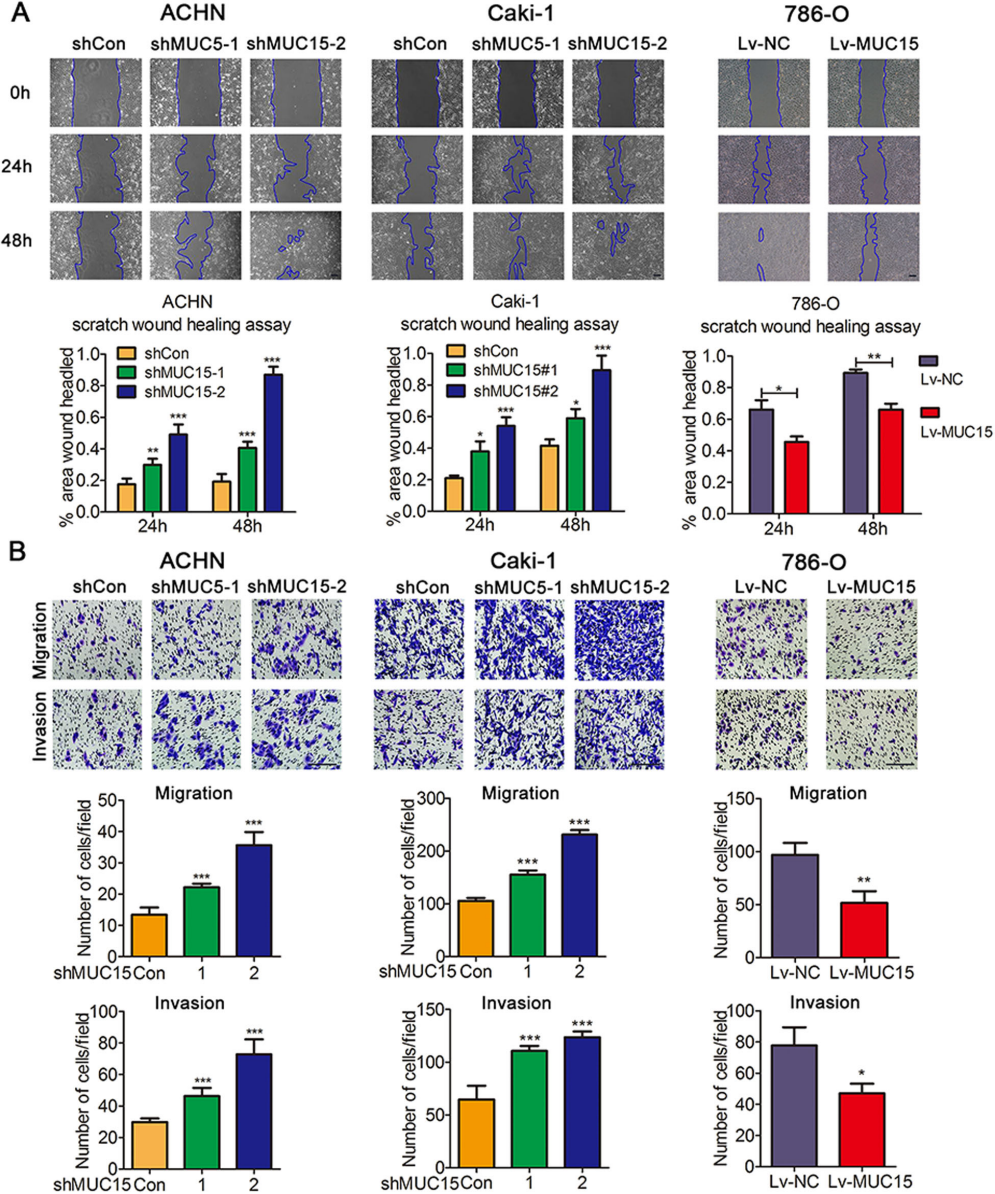
Effects of MUC15 in cell migration and invasion of RCC cell lines. **a** Representative images of wound healing in MUC15 Knock-down (KD) ACHN and Caki-1 sublines, MUC15 overexpressing (OV) 786-O sublines, quantification analysis was shown below (*N* = 3). **b** Representative pictures of Transwell migration and invasion in MUC15-KD ACHN and Caki-1 sublines, MUC15-OV 786-O sublines, quantification analysis was shown below (*N* = 3). The scale bar represents 100 μm (*P < 0.05, **P < 0.01, ***P < 0.001).

The sustaining proliferation and activating metastasis are vital aspects of tumor progression^17–19^, therefore, we aimed to explore these two important biological functions of MUC15 in RCC. For tumor cell proliferation, we did not observe any significant changes in MUC15 knocking-down ACHN or Caki-1 cells, and MUC15 overexpressed 786-O cells (Supplementary Fig. 2A–C). However, Knocking-down of MUC15 in ACHN and Caki-1 cells led to an increased wound healing rate, indicating that MUC15 inhibited the ability of migration in RCC cells. Inversely, the wound healing rate was inhibited in MUC15 overexpressed 786-O cells (Fig. 3a). Similarly, in Transwell migration and invasion assays as showed in Fig. 3b, knocking-down MUC15 could promote cell migration and invasion in ACHN and Caki-1 cells. In contrast, overexpression of MUC15 in 786-O cells restrained cell migration and invasion. Thus, we could get a conclusion that MUC15 suppressed RCC metastasis in vitro.

### MUC15 could regulate PI3K/AKT signaling and MMPs expression

**Fig. 4:**
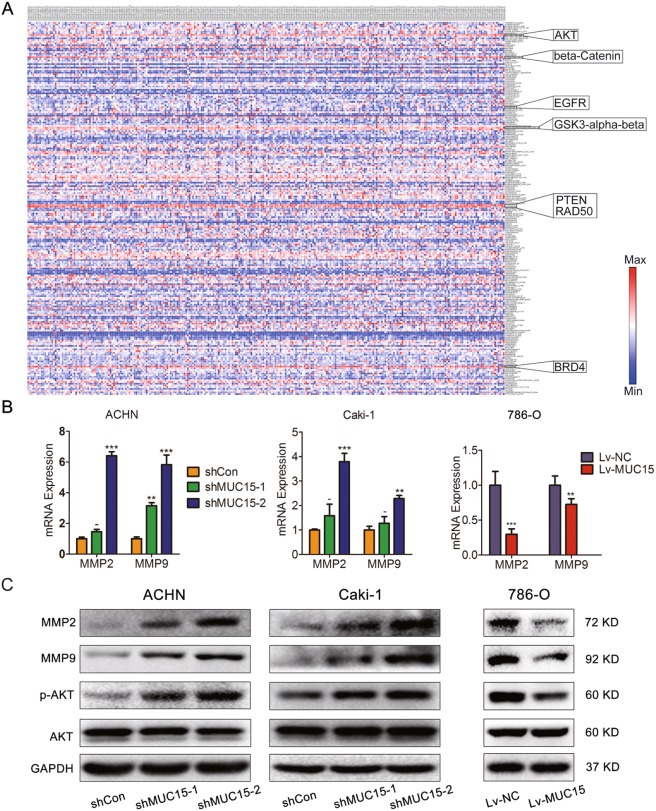
Effects of MUC15 in PI3K/AKT signaling and MMPs expression. **a** The heat map of protein expression of 278 RCC samples in TCGA database was analyzed and drawn by MORPHEUS. AKT, beta-Catenin, GSK3-alpha-beta, PTEN, RAD50, BRD4 protein expression level was elevated significantly in all tested proteins. **b** Real-time quantitative PCR analysis of MMP2 and MMP9 mRNA expression level in MUC15-KD ACHN and Caki-1 sublines, MUC15-OV 786-O sublines (*N* = 3). **c** Western blot analysis of MMP2, MMP9 and PI3K/AKT signaling in MUC15-KD ACHN and Caki-1 sublines, MUC15-OV 786-O sublines. 18S was applied as the endogenous control for quantitative real-time RT-PCR, and GAPDH was used as a loading control for western blotting assay (*N* = 3) (*P < 0.05, **P < 0.01, ***P < 0.001).

In the previous study, it has been proved that Matrix metalloproteinases (MMPs) are associated with the cancer progression^20^. Traditionally, MMPs played roles in matrix remodeling, particularly in cancer invasion and angiogenesis^21^. To further investigate the underlying mechanism of MUC15 suppressing RCC metastasis, we examined the changes of MMPs family in RCC cell lines with modification of MUC15 expression levels. Indeed, as shown in Fig. 4, both in mRNA and protein levels, we observed an increase of MMP2 and MMP9 in ACHN and Caki-1 cells with MUC15 knock-down, but decrease of MMP2 and MMP9 in 786-O cells with MUC15 overexpression. At the same time, after analyzing the mRNA expression of MMP2 and MMP9 in TCGA database, there was a negative correlation between MUC15 and MMP9 expression (Supplementary Fig. 2D, E).

Furthermore, we explored the upstream signaling to regulate the expression of MMP2 and MMP9. As showed in Fig. 4a, according to the heat map of protein expression of 278 RCC samples in TCGA database, AKT, beta-Catenin, GSK3-alpha-beta, PTEN, RAD50, BRD4 protein expression level was elevated significantly in all tested proteins, so we considered that those molecules played vital roles in RCC. Activation of PI3K/AKT has been proven to regulate tumor cell migration and invasion through degradation of MMPs-mediated matrix or activation of various transcription factors^22,23^. Therefore, we examined whether MUC15 modulated PI3K/AKT pathways to affect cell migration and invasion of RCC cells. Indeed, we observed an increased AKT phosphorylation in ACHN and Caki-1 cells with MUC15 knock-down, but decreased in 786-O cells with MUC15 overexpression, which indicated a negative regulation of AKT activation by MUC15 (Fig. 4b, c). However, we failed to observe a significant change of phosphorylation of EGFR, GSK3-beta and expression of beta-Catenin after knock-down of MUC15 in ACHN cells (Supplementary Fig. 2F). Also, there was no positive correlations between phosphorylation of EGFR and MUC15 mRNA expression in RCC specimens from TCGA (Supplementary Fig. 2G–I).

### MUC15 repressed RCC cell migration and invasion via PI3K/AKT signaling

**Fig. 5:**
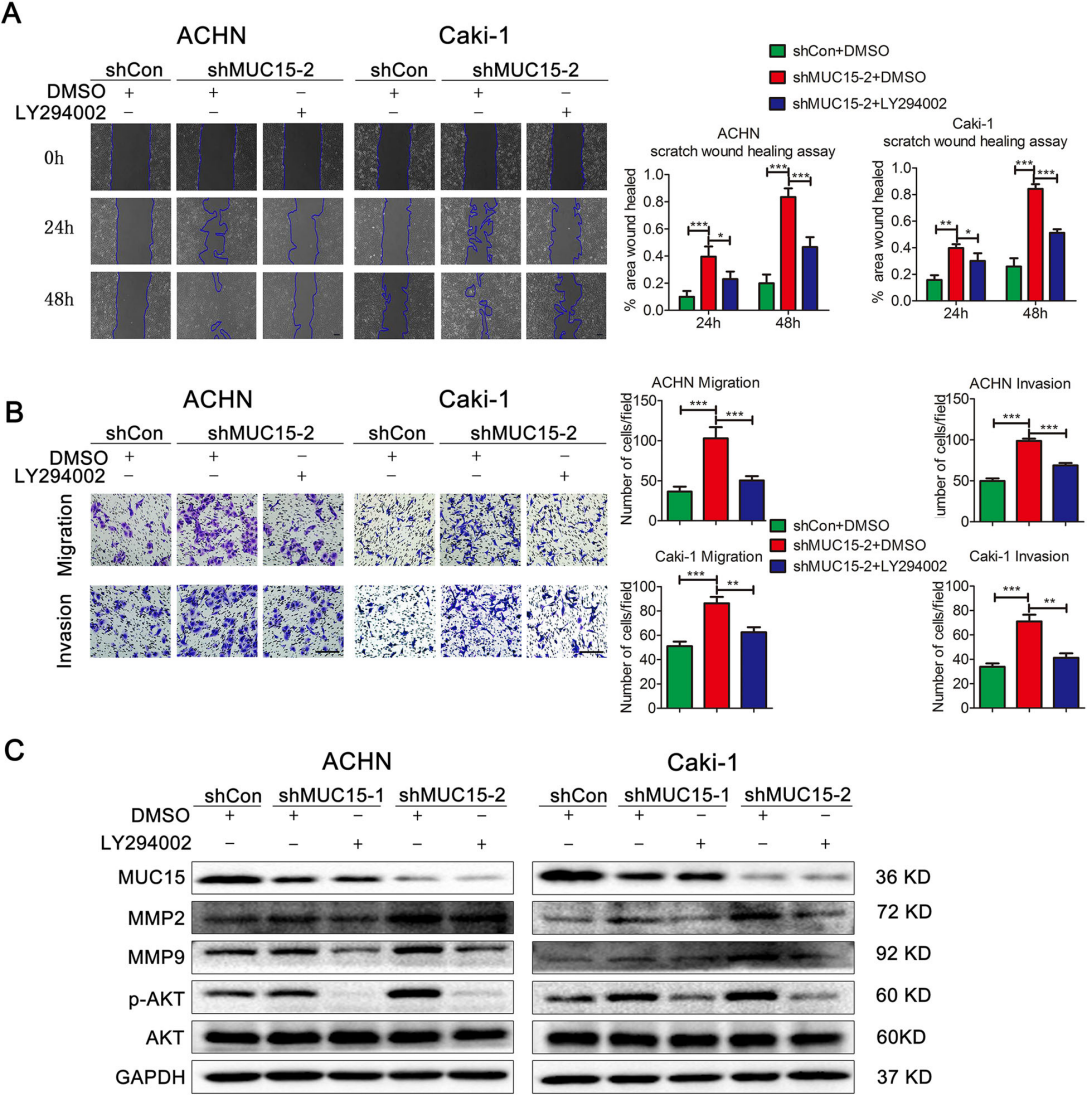
MUC15 modulated RCC cell migration and invasion via PI3K/AKT signaling. **a** Representative images of wound healing in MUC15-KD ACHN and Caki-1 sublines treated with PI3K inhibitor LY294002, quantification analysis was shown on the right (*N* = 3). **b** Representative pictures of Transwell migration and invasion in MUC15-KD ACHN and Caki-1 sublines treated with LY294002, quantification analysis was shown on the right (*N* = 3). **c** Western blot analysis of MMP2, MMP9 and PI3K/AKT signaling in MUC15-KD ACHN and Caki-1 sublines treated with LY294002. GAPDH was used as a loading control (*N* = 3). The scale bar represents 100 μm. (*P < 0.05, **P < 0.01, ***P < 0.001).

To confirm the role of PI3K/AKT pathways in RCC metastasis, the PI3K inhibitor LY294002 was applied in RCC sublines with modification of MUC15 expression level. Indeed, in ACHN and Caki-1 cells with MUC15 knock-down, we found that the increase of wound healing rate was partly reversed at 24 h and 48 h after LY294002 treatment (Fig. 5a). At the same time, in Transwell migration and invasion assays as shown in Fig. 5b, LY294002 could also abolish the increase of cell migration and invasion. Consistently, as shown in Fig. 5c, the upregulation of MMP2, MMP9 also were abolished after LY294002 treatment.

### MUC15 inhibited RCC cells distant metastasis in vivo

**Fig. 6:**
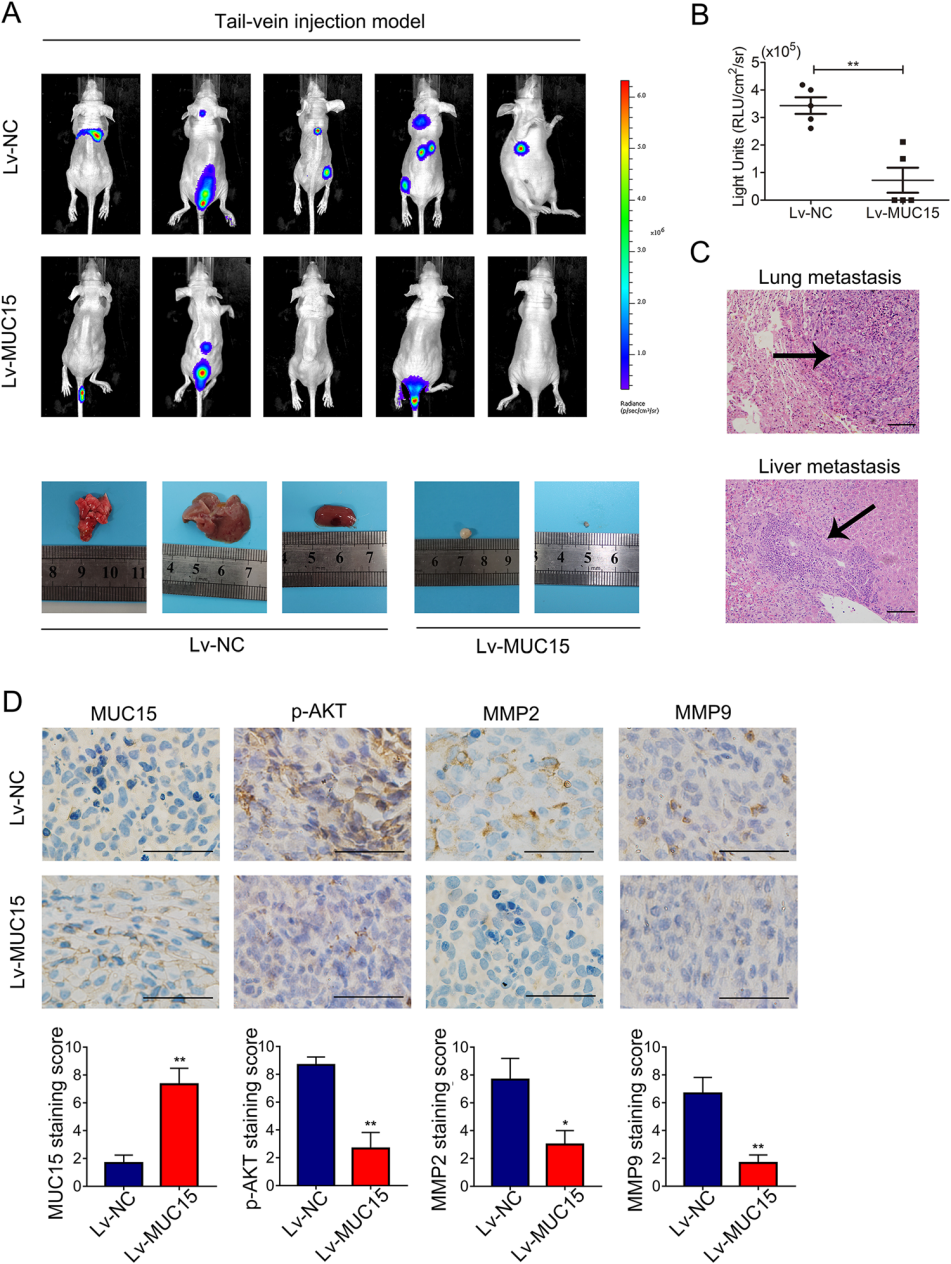
MUC15 modulated RCC cells distant metastasis in vivo. **a** BLI images of athymic BALB/c nude mice implanted with 786-O/NC and 7860/MUC15 cells by tail-vein injection, the images of lung, liver and adjacent metastasis were showed below (*N* = 5). **b** Quantification of light emission for metastases in mice (*N* = 5). (**P < 0.01). **c** Representative images of Hematoxylin- eosin (HE) staining of lung and liver metastatic tumor (*N* = 3). **d** Representative images of immunohistochemistry staining of MUC15, p-AKT, MMP2 and MMP9 in distant or adjacent metastatic tissues from 786-O/NC and 7860/MUC15 cells (*N* = 3). The scale bar represents 50 μm.

To verify the tumor-suppressive role of MUC15 in RCC in vivo, we established the tail-vein injection metastasis model using 786-O sublines in nude mice. As shown in Fig. 6a, we observed multiple metastases of lung, liver, brain, abdominal cavity and spinal column when 786-O negative control (NC) cells were injected via tail vein, meanwhile only few metastases were observed in spinal column when MUC15-overexpressing 786-O were injected, indicating that overexpression of MUC15 in 786-O cells dramatically abolished the incidence of distant metastasis (Fig. 6b, *P* < 0.01). Also, we compared the expression of p-AKT, MMP2 and MMP9 in the distant metastatic tissues by IHC staining. In consistent with our in vitro results, 786-O tumors with higher MUC15 expression showed lower p-AKT, MMP2 and MMP9 staining compared with NC (Fig. 6d). All these results indicated that MUC15 could inhibit RCC metastasis in vivo.

## Discussion

Patients with metastatic RCC usually have a very poor survival in clinic. Nowadays, although many new targeted or immunotherapy drugs have been used for treatment, they will inevitably acquire drug resistance^24^. Therefore, it is necessary and important to understand more molecular mechanisms of RCC metastasis and drug resistance.

MUC15 is one of cell membrane-associated mucins that are high-molecular-weight proteins in multiple types of epithelial^25,26^. The protein of MUC15 is predicted to contain a highly conserved cytoplasmic tail, an extracelluar domain and a small transmembrane domain^25,27^, which indicated that MUC15 maybe play a vital role in cell signal transduction. Shyu et al.^12^ firstly studied the function of MUC15 in human placenta and demonstrated that overexpression of MUC15 substantially inhibited matrigel invasion of trophoblast-like cells JAR and JEG-3. Huang et al.^13^ showed that mRNA and protein expression of MUC15 were significantly higher in colorectal tumors compared with their normal counterparts, furthermore, in vitro and in vivo experiments showed that overexpression of MUC15 could activate extracellular signal-regulated kinase 1/2 and promote the progression of human colon cancer cells. In thyroid carcinoma, Nam et al.^15^ revealed the association between high MUC15 expression and aggressive malignant potential, which indicated that MUC15 may serve as a prognostic marker and potential therapeutic target. Moreover, Choi et al.^16^ demonstrated that MUC15 could promote tumor progression and cancer stemness via GPCR/ERK and integrin-FAK signaling pathways. However, MUC15 plays a opposite role in hepatocellular carcinoma^11^. MUC15 mRNA and protein Levels were obviously lower in hepatocellular cancer than normal tissues, and patients with lower MUC15 had shorter overall survival and disease-free survival. Furthermore, MUC15 could restrain the aggressive behavior by inhibiting dimerization of EGFR and PI3K–AKT signaling in vitro and in vivo.

In our study, we confirmed that MUC15 was notably decreased in RCC compared to normal tissue. However, the mRNA and protein expression in normal renal cell line were not highest compared to renal cancer cell lines, and this may be due to the heterogeneity of cell lines. Furthermore, we demonstrated that MUC15 could suppress cell migration and invasion of RCC by the strategy of knocking-down or overexpressing MUC15. Also, we found that knocking-down MUC15 could active the PI3K/AKT signaling by increasing the AKT phosphorylation and subsequently increase the mRNA and protein expression of MMP2 and MMP9. However, we failed to detect a significant change of EGFR phosphorylation in RCC cell lines, indicating that the function of MUC15 in different cancer type may be cell specific. Taken together, MUC15 could be a potential novel potential prognostic marker and drug target for RCC diagnosis and treatment.

## Materials and methods

### Cell culture and reagents

Human RCC cell lines 769-P, 786-O, ACHN were obtained from the American Type Culture Collection (ATCC). Human RCC cell lines Caki-1, OS-RC-2 were purchased from National Platform of Experiment Cell Resources for SciTech (Beijing, China), and Human RCC cell lines RCC42 was kindly presented by Dr. JerTsong Hsieh (University of Texas Southwestern Medical Center, Dallas, TX, USA). All cell lines were maintained in RPMI1640 medium with 10% fetal bovine serum (FBS) at 37 ˚C and 5% CO2. Cells used in experiments were in good condition without mycoplasma contamination. LY294002 was obtained from Selleckchem (Houston, TX, USA). The antibodied were as follows: MUC15 (Rabbit, Sigma-Aldrich, St Louis, MO, USA), β-actin and GAPDH (mouse, Kangchen Bio-tech, Shanghai, China), AKT, p-AKT, p-EGFR, p-GSK-3β, β-Catenin and MMP2 (Rabbit, Cell Signaling Technology, Danvers, MA, USA), MMP9 (Rabbit, abcam, Inc., Cambridge, Britain).

### Clinical specimens and immunohistochemistry

All the RCC and nomal tissues were collected from the Department of Urology, The first Affiliated Hospital of Xi’an Jiaotong University, Xi’an, China. The Collection of clinical samples was approved by the Ethical Committee of Hospital and informed consent was obtained from all patients. The tissue sections were deparaffinized in 60 °C for 4 h and immersed in xykene quickly, rehydrated in a series of grade alcohols. After washing 3 times by 5 min once with PBS buffer (exclusive of potassium), tissue sections were subjected to 5-min pressure cooker antigen retrieval methods, 10-min of quenching endogenous enzyme. Next, the primary antibody was incubated at 4 °C overnight, and Dako Cytomation EnVision-HRP reagent was incubated for 30 mins at room temperature after washing with PBS. Then, adding diaminobenzidine (DAB) to detect the signals and hematoxylin to stain nucleus. The result was evaluated by the intensity of staining (0, 1, 2+, 3+) and the percentage of positive cells separated by 0 (0%), 1 (1–25%), 2 (26– 50%), 3 (51–75%) and 4 (76–100%). The product of two scores reveals the staining level negative (0 score), weak (1–4 score), moderate (5–8 score) and strong (9–12 score).

### Plasmid transfection and lentiviral infection

MUC15 short hairpin RNA (shRNA) was applied to knocked-down MUC15 in RCC cell lines. The plasmids LVRU6GP contained the shRNA of MUC15 were purchased from GeneCopoeia (Guangzhou, China), then lentiviral system pLKO.1 (Oligoengine, Seattle,WA) was constructed to infect cells. For overexpression of MUC15, the lentiviral system EX-E2664-Lv201 (GeneCopoeia, Guangzhou, China) containing full-length MUC15 was used. After 48 h of transfection with 8 μg/ml polybrene following the manufacturer’s instructions, the cells were harvested for next experiments.

### Wound healing assay

RCC cell lines ACHN and Caki-1 with MUC15 knock-down or 786-O with MUC15 overexpression were planted in 6-well plate with cross marker lines on the back. Using a 200 µl pipette tip to cut out the artificial wounds, then the wounds healing was observed by inverted microscope after 12 and 24 h at the same position. The software Image Pro Plus 6.0 (Media Cybernetics company, USA) was applied to analyze the percentage of wounds healing area.

### Transwell assay

For transwell migration assay, cells were harvested and 4 × 10^4^ cells in 300 µl serum-free RIPA-1640 were added into the upper chamber with 8-µM pore polycarbonate membrane flters (Millipore, USA). For invasion assay, 8 × 10^4^ cells in 300 µl serum-free RIPA-1640 were added into the upper chamber inserts with Matrigel (BD Biosciences, USA) flated for 4 h in advance. 1 ml medium containing 10% FBS was added in lower chamber. After incubation for 24 h or 36 h, the Transwell inserts were fixed with 4% paraformaldehyde for 15 min and stained with 0.1% crystal violet for 15 min at room temperature. Then, the number of cells was counted in three random fields in 100× magnification inverted light microscope.

### Real-time RT-PCR

The RNAfast 200 reagents (Fastagen Biotechnology, Shanghai, China) was used to extract the total cellular RNA. After quantitating by absorbance at 260 nm, the RNA sample was transcribed reversely by PrimeScript RT Master Mix (Takara Bio, Dalian, China) according to the suggested system. Then the quantitative PCR was accomplished by SYBR-Green PCR Master Mix (Takara Bio, Dalian, China) with specific primers as follows: MUC15, F: TATTCACTTCTATCGGGGAGCC, R: GGGAATGACTCGCCTTGAGAT; MMP2, F: GATACCCCTTTGACGGTAAGGA, R: CCTTCTCCCAAGGTCCATAGC; MMP9, F: GGGACGCAGACATCGTCATC, R: TCGTCATCGTCGAAATGGGC; 18S, F: CAGCCACCCGAGATTGAGCA, R: TAGTAGCGACGGGCGGTGTG.

### Western blot analysis

The cells lysates were obtained with RIPA buffer (50 mM Tris, PH 8.0,150 mM NaCl, 0.1% SDS, 1% NP40 and 0.5% sodium deoxycholate) containing proteinase inhibitors (1% inhibitors cocktail and 1 mM PMSF) (Roche Applied Science, Germany) for 10 min in ice and centrifuged at 12,000 *g* for 15 min at 4 °C. The samples were separated by 12% SDS-PAGE and transferred to polyvinylidene fluoride (PVDF) membranes. After blocking with 5% non-fat milk, the membranes were incubated with primary antibody at 4 °C overnight. Next, the membranes were washed with TBST buffer for 3 times and incubated with peroxidase-conjugated secondary antibody for 1 h at room temperature. After washing with TBST buffer for 3 times again, the membranes were visualized with an ECL chemiluminescent detection system (Bio-rad, USA).

### Tail-vein cancer metastasis model

Nude tail-vein injection model was performed as the cancer metastasis model based on previous studies^28^. Female athymic BALB/c nu/nu mice about 4–6 weeks old were applied to the experiment based on the document of the ethical committee of Xi’an Jiaotong University. Ten mice were randomly divided into two groups by random scale as Lv-NC and Lv-MUC15. RCC cell line 786-O with MUC15 overexpression or negative control were maintained and harvested, 2 × 10^6^ cells were suspended with serum-free RIPA-1640 and injected via the tail vein with insulin needle. After 6 weeks, D-luciferin substrate (Biosynth, Naperville, IL, USA) in PBS with 450 mg/kg was injected into abdominal cavity, 15–20 min later, bioluminescence imaging (BLI) was performed to detect the distant metastases in the lung and other organs after mice were anesthetized.

### Bioinformatics and statistical analysis

The RCC public datasets GSE6344 and GSE781 were downloaded from NCBI GEO database. The MUC15 expression data based on RNA-sequence and protein expression data were acquired from cBioPortal *(*www.cbioportal.org*)* for The Cancer Genome Atlas (TCGA)^29^. The samples without data were excluded from the analysis. The mRNA expression data was analyzed and performed by GraphPad Prism version 7.0 software (GraphPad Software, USA). The heat map of protein expression of 278 RCC samples was analyzed and drawn by web tool MORPHEUS (https://software.broadinstitute.org/morpheus/). All the statistical analyses were performed by SPSS 22.0 software. All data were reported as mean ± SD of triplicate experiments, and the differences between two groups were compared by the two-tailed Student’s (*t*-test) or one-way analysis of variance. *P < 0.05 was considered statistically significant.

## Supplementary information

Supplementary Fig 1

Supplementary Fig 2

Supplemantary information
