# Supplementary figures and images for: MUC15 inhibits cancer metastasis via PI3K/AKT signaling in renal cell carcinoma
Source: Cell Death Dis. 2020 May 7;11(5):336. doi: 10.1038/s41419-020-2518-9 (PMC7205982; doi:10.1038/s41419-020-2518-9)

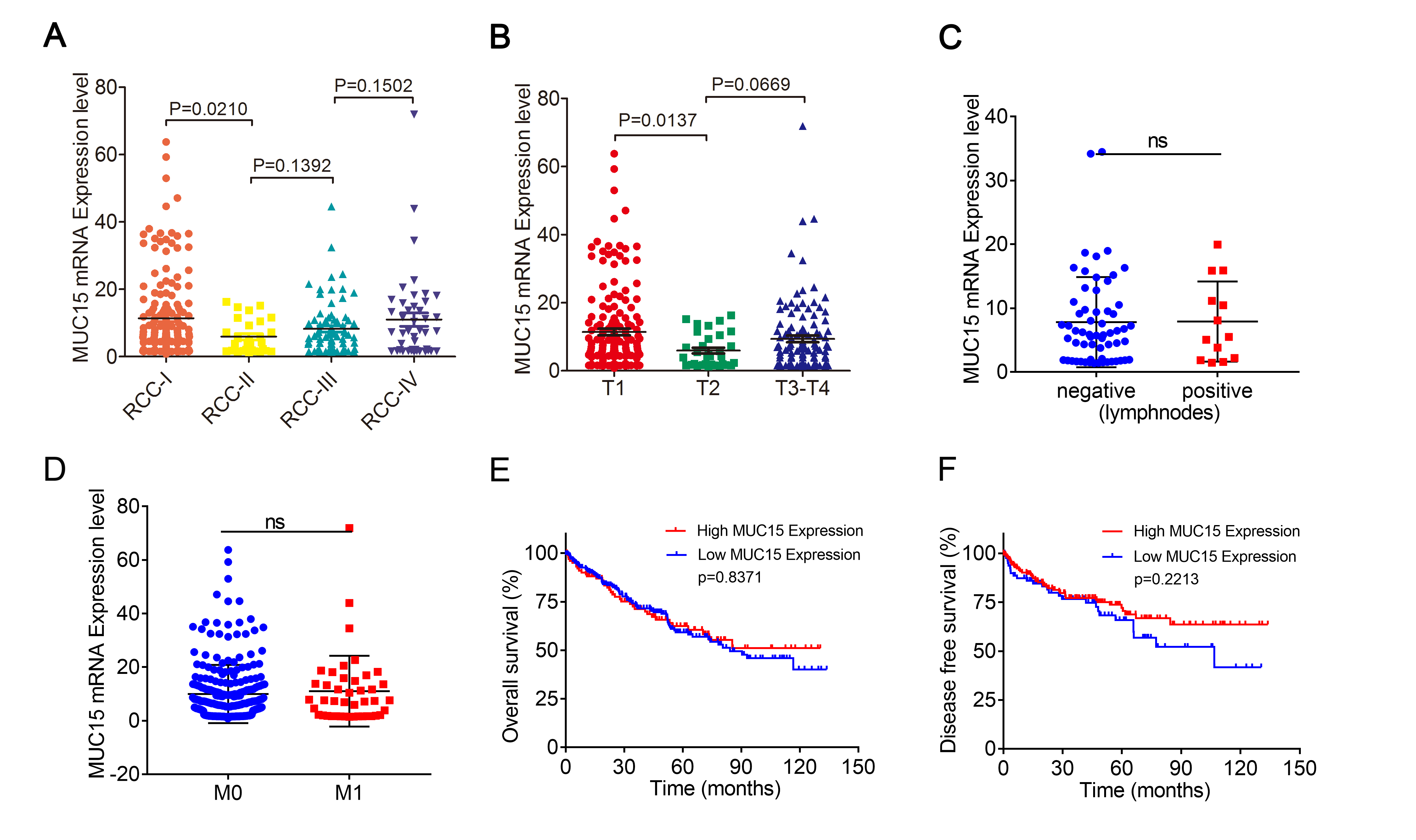

Supplement: Supplementary file 1 — Supplementary Fig 1 [file 41419_2020_2518_MOESM1_ESM.tif]

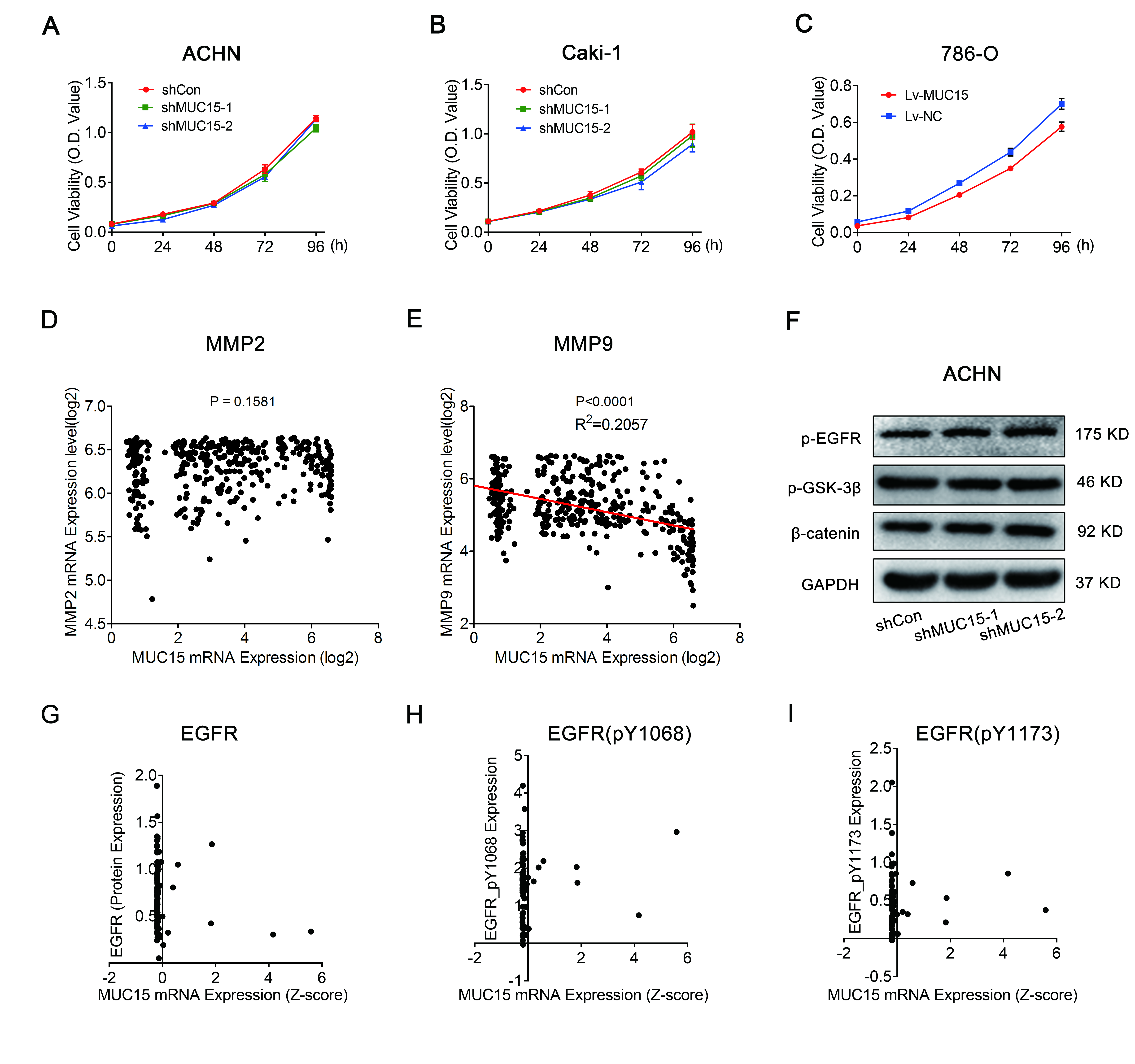

Supplement: Supplementary file 2 — Supplementary Fig 2 [file 41419_2020_2518_MOESM2_ESM.tif]
