# Supplementary material for: MUC15 inhibits cancer metastasis via PI3K/AKT signaling in renal cell carcinoma
Source: Cell Death Dis. 2020 May 7;11(5):336. doi: 10.1038/s41419-020-2518-9 (PMC7205982; doi:10.1038/s41419-020-2518-9)
Supplement: Supplementary file 3 — Supplemantary information [file 41419_2020_2518_MOESM3_ESM.doc]

**Supplementary Fig.1 Expression of MUC15 in RCC tissues and correlation with overall survival of patients.**

A, B, C and D, MUC15 mRNA expression in human RCC tissues with different grades (I-IV) and stages (T1-T4, lymph nodes positive or negative, M0 and M1) from TCGA database (n=313). E and F, Kaplan-Meier analysis of overall survival (OS) and disease free survival (DFS) in RCC patients with high (n =103) and low (n =203) MUC15 mRNA expression from TCGA database.

**Supplementary Fig.2 Effects of MUC15 in cell proliferation and the correlation between MMPs and MUC15.**

A, B and C, Cell viability reveals the proliferation of ACHN and Caki-1 cells with MUC15 knocked-down or 786-O with MUC15 overexpression (N = 3). D and E, The correlation between MMP2 (or MMP9) and MUC15 in mRNA expression level based on TCGA database (n = 315). F, Western blot analysis of p-EGFR (TYR1045), p-GSK-3β (ser9), β-catenin protein expression level in human renal cancer cell line ACHN with MUC15 knock-down, GAPDH was used as a loading control (N = 3). G, H,I, The correlation between the protein expression of EGFR, EGFR (pY1068) or EGFR (pY1173) and MUC15 mRNA expression based on TCGA database (n = 278).
